# Enhanced stability of freestanding lipid bilayer and its stability criteria

**DOI:** 10.1038/srep38158

**Published:** 2016-12-16

**Authors:** Dae-Woong Jeong, Hyunwoo Jang, Siyoung Q. Choi, Myung Chul Choi

**Affiliations:** 1 KAIST, Department of Bio and Brain Engineering, Daejeon, 34141, Korea; 2 KAIST, Department of Chemical and Biomolecular Engineering, Daejeon, 34141, Korea

## Abstract

We present a new strategy to dramatically enhance the stability of freestanding lipid bilayers. We found that an addition of a water in oil emulsion stabilizer, SPAN 80 to a solvent phase guarantees nearly millimeter-scale stable freestanding lipid bilayers. The water permeability, bilayer area, contact angle, and interfacial tension were measured as a function of time and SPAN 80-to-lipid weight ratio (Φ_SPAN 80_) with several different solvents. Surprisingly, the SPAN 80, instead of remaining in the bilayer, was moved out of the bilayer during the bilayer formation. Also we studied the effect of solvent on freestanding bilayer formation, and found that squalene was the only solvent that was not incorporated into the bilayer. The regime of stable bilayer formation was experimentally determined to be 3/1 < Φ_SPAN 80_ < 15/1, and we suggest general stability criteria for bilayer formation. This technique and the suggested stability criteria can be potentially helpful to many model membrane-based researches in life sciences, physical sciences and biomedical engineering fields.

Engineering artificial model cell membranes, such as lipid vesicles^1^,^2^, supported lipid bilayers^2^,^3^,^4^,^5^, and freestanding lipid bilayers^6^,^7^,^8^,^9^,^10^,^11^,^12^,^13^,^14^,^15^,^16^,^17^,^18^,^19^,^20^,^21^,^22^,^23^,^24^, is an important issue in physical science, life science and biomedical engineering^25^,^26^. Such model membranes can provide a useful *in vitro* platform for studying a variety of biological problems related to cell membranes^27^,^28^. Of those, freestanding lipid bilayers have advantages over other techniques in that both sides of the lipid bilayer can be under the control of various physicochemical parameters, including ionic strength and pH, chemical and biological molecules. In particular, freestanding lipid bilayers with large area (≥several hundred μm) and horizontally planar geometry would enable important problems to be tackled such as the direct visualization of structures, phase behaviors^29^,^30^,^31^,^32^, dynamic behaviors^33^, and interactions of complex systems (e.g. membrane proteins, lipid rafts, nanoparticles, other vesicles) in a simplified model cell membrane^14^,^15^,^18^,^24^.

The challenge in the formation of freestanding lipid bilayers is their poor stability. Accordingly, a lot of new fabrication methods for stable freestanding lipid bilayers have been introduced^6^,^7^,^8^,^9^,^10^,^11^,^12^,^13^,^14^,^15^,^16^,^17^,^18^,^19^,^20^,^21^,^22^. Recently, an excellent technique, termed as droplet interface bilayers (DIBs) was developed^18^,^19^,^20^,^21^,^22^,^23^ where two lipid-coated water droplets immersed in solvent bring together to form a stable freestanding lipid bilayer with large area. The DIBs can be used for imaging experiments and easily incorporate membrane proteins with simple fabrication process. Notably, Wallace and coworkers recently developed a model lipid bilayer by placing a lipid-coated droplet on top of a lipid-coated agarose gel to enhance its stability, while taking advantage of horizontally planar geometry for imaging experiments^21^,^22^.

In this paper, we introduce a new strategy to dramatically enhance stability of DIBs by using water in oil (W/O) emulsion stabilizer, SPAN 80. Our lipid bilayer is also horizontally planar as others^21^,^22^, but it is formed on top of water instead of agarose gel. Figure 1 shows the schematic illustration of how a freestanding DIB is formed, and provides the corresponding microscopy images of freestanding lipid bilayer formation, monitored by side-view microscope. We prepared a planar interface between water and squalene in which lipids and SPAN 80 were dissolved. The lipids and SPAN 80 are spontaneously adsorbed to the interface, forming a planar monolayer. A water droplet with a size range of 100–500 μm in diameter was introduced into the squalene, where the other monolayer was formed at the spherical water droplet. As a lipid coated water droplet approaches a plane interface, the solvent phase between the interfaces drains out and the two monolayers undergo a “zipping” process during which the two monolayers adhere to each other^14^,^15^,^16^,^17^,^18^,^19^,^20^,^21^,^22^,^23^,^34^, resulting in a horizontally planar freestanding lipid bilayer. Our control parameter was the SPAN 80-to-lipid weight ratio Φ_SPAN 80_ (with a constant lipid concentration of 1 mg/ml), which determines the squalene/water interfacial tension.

## Results and Discussion

Interestingly, in all our experiments, the freestanding bilayers remained stable at least for several days without changes in the bilayer area or contact angle for an appropriate range of Φ_SPAN80_ whereas a droplet immediately coalesced for too low Φ_SPAN80_. This implies that the SPAN 80 dramatically enhances the stability of freestanding lipid bilayer^18^,^20^,^21^,^22^,^23^. Such enhancement in stability of the freestanding bilayer can be explained by the role of SPAN 80 during the impact of the two monolayers which include the following. First, it modifies the spontaneous curvature; its hydrophobic tail is bulky relative to the hydrophilic head, which induces negative spontaneous curvature. For the bilayer with low stability, a transient pore, which is hydrophilic pore through the bilayer with highly positive curvature, is formed before merging of droplet into sub-phase water. The SPAN 80 with negative curvature plays a critical role for preventing the formation of transient pore to stabilize bilayer^35^. Second, it modifies the interfacial tension. As Φ_SPAN80_ increases, the interfacial tension decreases, thus it reduces the energetic benefit of droplet coalescence^35^. We also checked other surfactants such as oxidized squalene and docosahexaenoic acid (DHA) that have similar molecular shapes significantly enhance the stability of lipid bilayers. In the previous study, this level of stability was achieved only when the limited kinds of lipid (e. g. 1,2-diphytanoyl-sn-glycero-3-phosphocholine, DPhPC) with exceptionally bulky tail or solvent (e. g. hexadecane) remaining in the bilayer after zipping process are used, and this result indicates that the SPAN 80 dramatically enhances stability of the freestanding lipid bilayer^18^,^20^,^21^,^22^,^23^.

**Figure 2:**
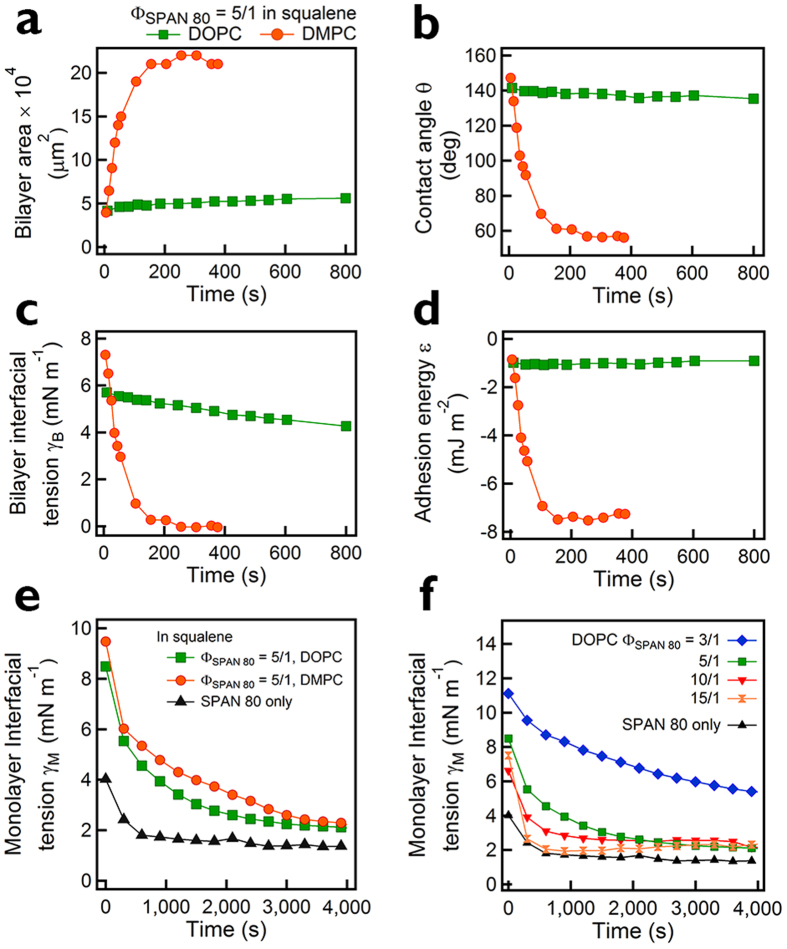
For the freestanding DOPC and DMPC bilayers in squalene at Φ_SPAN80_ = 5/1, plots of Bilayer area (**a**), Contact angle (**b**), Interfacial tension (**c**), and Adhesion energy (**d**) vs. time. The t = 0 is when the bilayers are formed. (**e**) Monolayer interfacial tension vs. time for DOPC and DPPC at SPAN 80-to-lipid weight ratio (Φ_SPAN80_) of 5/1, and for SPAN 80 only. (**f** ) Monolayer interfacial tension vs. time as a function of Φ_SPAN80_.

It was expected that our bilayers would be composed of a mixture of lipid and SPAN 80. However, we surprisingly found that SPAN 80 is likely to be moved out of the bilayer during/after the bilayer formation. To systematically verify the removal of SPAN 80 from the bilayer, we measured the bilayer area, contact angle, bilayer tension, and adhesion energy of two monolayers of dimyristoylphosphatidylcholine (DMPC) and dioleoylphosphatidylcholine (DOPC). We performed all of our experiments at 25 °C where both lipids exhibit a liquid disordered phase^36^. Figure 2(a,b) is the plot of the bilayer area and the contact angle of the freestanding bilayer as a function of time. At t = 0, the bilayer area is of the same diameter d ≈ 220 μm for both DOPC and DMPC. For DMPC, a drastic change in the bilayer area and the contact angle (*θ*) occurs at t < 200 sec, followed by the constant values d = 523 μm and *θ* = 56°, whereas for DOPC, the bilayer area and contact angle remain unchanged. Figure 2(c) shows the interfacial tension of the bilayer γ_B_ of DOPC and DMPC at Φ_SPAN80_ = 5/1. The bilayer interfacial tension is defined as the sum of the interfacial tension of monolayers on droplet surface and planar interface: Young’s equation *γ*_B_ = *γ*_M_ (1 − cos *θ*), where *γ*_M_ and *γ*_B_ are the interfacial tensions of the monolayer and the bilayer, respectively. Monolayer interfacial tension was measured by using a pendant drop technique as shown in Fig. 2(e) and (f)^37^. Both DOPC and DMPC have similar *γ*_M_, but *γ*_B_ of DOPC (4.3–5.7 mN/m) is far greater than *γ*_B_ of DMPC (∼0 mN/m at t > 200 sec). Figure 2(d) shows the adhesion energy per unit area, *ε* = *γ*_B_ − 2*γ*_M_ (in J/m^2^), of the DOPC and DMPC bilayer at Φ_SPAN80_ = 5/1. The equilibrium adhesion energy is −1.0 mJ/m^2^ (DOPC) and −7.4 mJ/m^2^ (DMPC).

The increase in the adhesion energy over time suggests that the bilayer composition changes after bilayer formation. When only SPAN 80 was used without any lipid, no adhesion was observed, implying that zero adhesion exists between SPAN 80 molecules. Therefore, to maximize the adhesion (to lower the energy), lipid molecules should go into the bilayer, excluding SPAN 80 out of the bilayer. At the same time, this demixing process of lipid and SPAN 80 results in an entropic penalty, more specifically, the entropy of mixing. In other words, the competition between adhesion energy and the entropy of mixing determines the distribution of SPAN 80. For the DOPC and DMPC bilayer at Φ_SPAN80_ = 5/1, the estimated entropic penalty of SPAN 80 is at most in the same order of magnitude as the energetic gain obtained by introducing more lipids in the bilayer region (see Supplementary Figure S3). Therefore, the decrease in the bilayer interfacial tension for DMPC at an early stage (t < 200 sec) in Fig. 2(c) supports that SPAN 80 is removed from the bilayer, to increase adhesion between the two monolayers, as seen in Fig. 2(d). During this period, SPAN 80 is removed from the lipid bilayer and the interfacial tension of the DMPC bilayer drops into the plausible range, when compared with the bilayer rupture tension *γ*_br_ (DMPC) ≈ 2.7 mN/m (at least, the bilayer tension should be smaller than the rupture tension). *γ*_B_ of DOPC (4.3–5.7 mN/m) is also less than *γ*_br_ (DOPC) ≈ 10.2 mN/m^38^.

**Figure 3:**
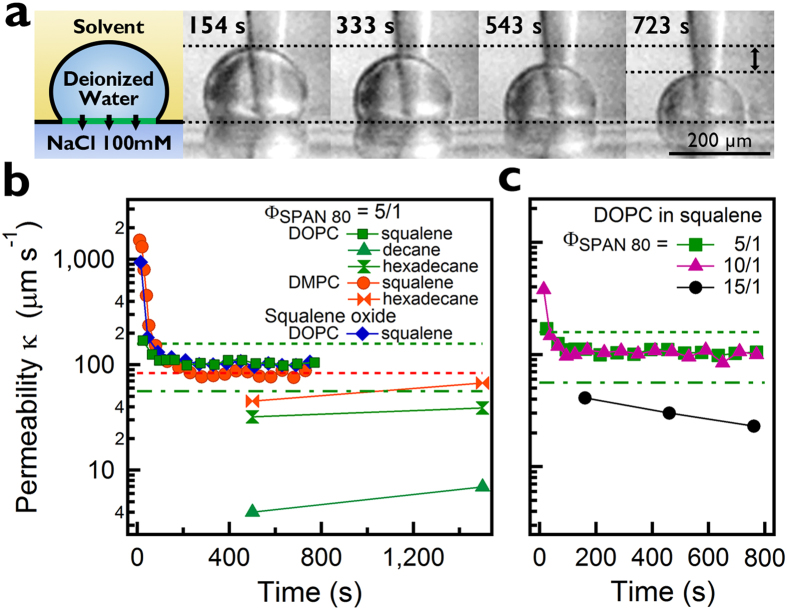
Water permeability of freestanding bilayers. (**a**) Side-view microscopy images show the decrease in the volume of droplet as water transports through the lipid bilayer by osmotic pressure gradient (100 mM NaCl at the bottom phase). (**b,c**) Water permeability vs. time, for DOPC and DMPC bilayer for different stabilizers (SPAN 80 and squalene oxide) and solvents (squalene, decane and hexadecane) (**b**) and for DOPC in squalene at different Φ_SPAN 80_ = 5/1, 10/1, 15/1 (**c**). The values from the previous works of *κ* = 83 ± 7.6 μm/sec for DMPC (dotted line in red), and *κ* = 56 ± 9 and 158 ± 5.8 μm/sec for DOPC (dash-dot and dotted lines in green) are shown^39^,^40^.

Another evidence that SPAN 80 is likely to be removed from the bilayer is shown in Fig. 3, the water permeability measurement. The 100 mM NaCl dissolved in the bottom water of the plane interface generates osmotic gradients across the bilayer, resulting in water transport through the lipid bilayer membrane (Fig. 3(a)). We measured the volume change in the water droplet as a function of time^19^,^20^. In Fig. 3(b), the water permeability of both the DMPC and DOPC bilayer at Φ_SPAN80_ = 5/1 decreases from 1521.3 μm/sec (DMPC) and 169.7 μm/sec (DOPC) to reach constant values of 83.0 ± 6.0 μm/sec (DMPC) and 103.6 ± 4.2 μm/sec (DOPC) after the bilayer formation. This equilibrium permeability is in good agreement with the previous measurements: 83 ± 7.6 μm/sec for DMPC, 56 ± 9 and 158 ± 5.8 μm/sec for DOPC^39^,^40^. The initial decrease in water permeability is consistent with the adhesion measurement and thus is most likely due to the process of removing SPAN 80 from the bilayer. Moreover, this initial decrease in water permeability is similarly shown for different stabilizer, squalene oxide, and values of the equilibrium permeability are almost identical (102.6 ± 6.0 μm/sec for squalene oxide) no matter what kind of stabilizer is used. This suggests that the freestanding bilayer at equilibrium might be composed of DOPC (or DMPC) lipid only.

The permeability result (Fig. 3(b)) also implies that our freestanding bilayer is squalene-free since this is consistent with the measurement for lipid vesicle that has no solvent in it. It is also widely known that squalene does not invade into bilayers or in between two monolayer leaflets^6^,^7^,^8^,^9^,^21^,^22^. Decane and hexadecane exhibit a lower permeability in comparison with squalene. A previous study reported that decane and hexadecane remain in the lipid bilayer after the formation of DIB^21^,^22^. When the bilayer contain a solvent such as decane or hexadecane, water molecules will cross the solvent layer in addition to the lipid bilayer, which results in drops in the water permeability. The interfacial tension of freestanding bilayer is 6.7–11.4 mN/m in decane, and 7.0–10.4 mN/m in hexadecane, which are significantly higher than the 4.3–5.7 mN/m in squalene (see Supplementary Figure S5). We note that the interfacial tensions of both decane and hexadecane are around the rupture tension of the DOPC bilayer (≈10.2 mN/m) in the absence of solvents.

In Fig. 3(c), the water permeability vs. time as a function of SPAN 80-to-DOPC weight ratio Φ_SPAN 80_, shows the concentration dependent role of SPAN 80 in the formation of the freestanding lipid bilayer. The bilayer zipping occurs at Φ_SPAN80_ > 3/1 (at Φ_SPAN80_ = 0 and 3/1, the bilayer formation fails, i.e. a droplet coalesces to water phase). For both Φ_SPAN80_ = 5/1 and 10/1, the same permeability *κ* = 98 μm/sec is measured. For Φ_SPAN80_ = 15/1, however, the permeability is 2.4–4.3 fold smaller (*κ* = 23–41 μm/sec). From this result, we conclude that the regime 3/1 < Φ_SPAN80_ < 15/1 is the condition under which a stable and solvent-free freestanding bilayer of DOPC in squalene can be formed.

**Figure 4:**
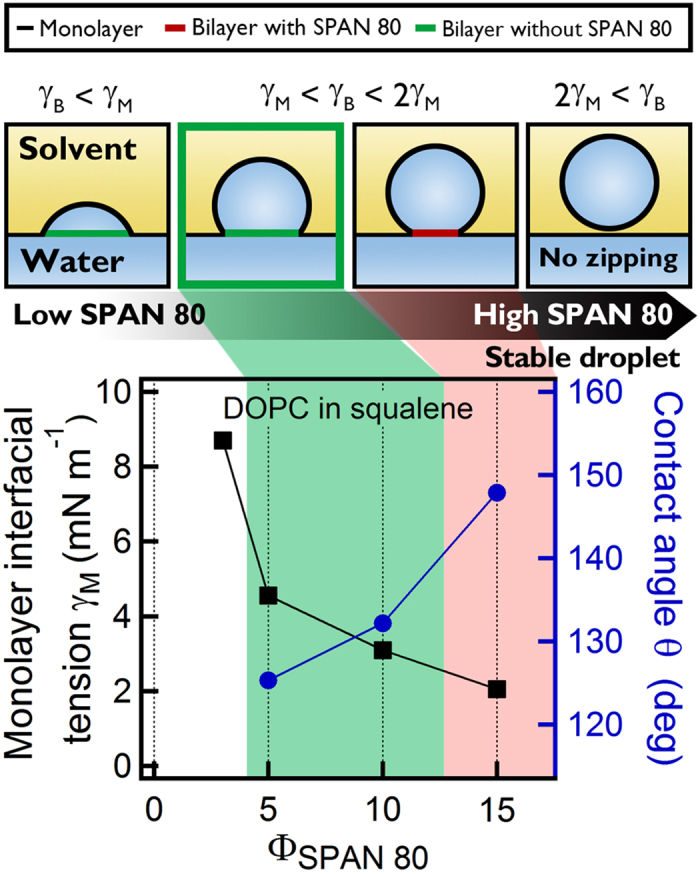
Stability criteria for freestanding bilayer formation. Monolayer interfacial tension for DOPC as a function of SPAN 80-to-lipid weight ratio Φ_SPAN80_ (symbol in black square, left axis). Contact angle is plotted in the right axis (in blue circle). The regime of stable and SPAN 80-free bilayer is shown in green. For higher Φ_SPAN80_, SPAN 80 is remained in the bilayer due to low adhesion, and for lower Φ_SPAN80_, SPAN 80 does not reduce the interfacial tension enough to form a stable bilayer.

Combining all the results above, we set up stability criteria for SPAN 80 stabilized bilayer formation (Fig. 4). At very low Φ_SPAN80_, SPAN 80 does not reduce the interfacial tension enough to form a stable bilayer, and thus coalescence immediately occurs as soon as a droplet is in contact with the planar surface. For sufficiently high Φ_SPAN80_, the interfacial tension is low enough for stable bilayers, exhibiting the successful zipping process with the intermediate contact angle between 90° and 180°. For very high Φ_SPAN80_ (in a case of 2*γ*_M_ < *γ*_B_), however, the contact angle reaches 180°, and the adhesion does not occur. Even if the contact angle does not reach 180°, too high Φ_SPAN80_ reduces adhesion between the two monolayers, and in this case, the entropy of mixing is too big to increase the adhesion, leaving some SPAN 80 in the bilayer. Moreover, the regulation of interfacial tension directly affects the three phase contact angle of the lipid bilayer: two lipid monolayers and a bilayer. The importance of the interfacial tension regulation is easily seen in the DMPC bilayer formation. For DMPC, the interfacial tension of the lipid bilayer is nearly zero. The DMPC monolayer interfacial tension is also low enough, so it appears to form a stable bilayer at first. However, the contact angle of the lipid bilayer changes over time, and eventually becomes very low (<60°), and the abrupt change at the kink seems to make the bilayer unstable. The stability of the DMPC bilayer becomes worse if the contact angle is very low. Therefore, to enhance the stability of the bilayer and to simultaneously obtain solvent-free and SPAN 80-free bilayers, there is an appropriate and optimum range of Φ_SPAN80_. Since different lipid species show different lipid bilayer interfacial tension, to form stable freestanding lipid bilayer this proper range of Φ_SPAN80_ will change. We also should note that previous DIBs use higher concentrations of lipids that might have an appropriate interfacial tension^18^,^19^,^20^,^21^,^22^,^23^.

## Conclusion

We demonstrated a new strategy to dramatically enhance stability of DIB with a large area, planar and solvent-free as well by using W/O emulsion stabilizer, SPAN 80. Surprisingly, SPAN 80 is most likely to be moved out of the bilayer, maximizing the adhesion of the lipid monolayers, and overcoming the entropy of mixing penalty. This removal of SPAN 80 was demonstrated by time-dependent adhesion and permeability experiments. We also showed that the freestanding bilayer fabricated by our technique is squalene-free as well. We finally suggested stability criteria for the SPAN 80 stabilized freestanding bilayer formation, involving the regulation of interfacial tension by controlling SPAN 80 concentration. This stabilization strategy can be universally applied to various freestanding bilayer formation techniques such as the conventional DIBs and the traditional black lipid membranes.

## Methods

Dimyristoylphosphatidylcholine (DMPC), dioleoylphosphatidylcholine (DOPC) and SPAN 80 are purchased. Squalene oxide is prepared by direct light exposure on squalene for four days with air contact. We use deionized water for all of our experiments. The imaging experiments were performed by using homebuilt side-view microscope. The sample of phospholipid (DMPC or DOPC) in chloroform is contained in glass vial and dried in vacuum. SPAN 80 dissolved in squalene is added into the dried phospholipid, and then sonicated for 30 minutes. We prepare a trough filled with water, and the phospholipid solution is placed on top of water to form a planar squalene/water interface. The glass capillary of 0.78/1.0 mm in inner/outer diameter respectively is tapered to 10 μm of diameter by a micropipette puller. The capillary is filled with water and then mounted to the micro-injector. The capillary tip is placed above the squalene/water interface. By applying a pressure of ~100 hPa, the droplet of ~300 μm diameter is introduced right above the planar interface. Both planar and droplet squalene/water interfaces are incubated for over 10 minutes for the adsorption of phospholipid and SPAN 80 monolayers, which are termed as planar monolayer and droplet monolayer, respectively. The droplet is moved toward the planar interface until the droplet gently touches the planar interface. After a few minutes of waiting, two monolayers undergo “zipping” process, in result, form the lipid bilayer between two water phases. The size of freestanding lipid bilayer can be controlled by adjusting the droplet size. Further details of monolayer interfacial tension measurement, water permeability measurement, and adhesion energy measurement are summarized in the Supplementary Information.

## Additional Information

**How to cite this article**: Jeong, D.-W. *et al*. Enhanced stability of freestanding lipid bilayer and its stability criteria. *Sci. Rep.* **6**, 38158; doi: 10.1038/srep38158 (2016).

**Publisher’s note:** Springer Nature remains neutral with regard to jurisdictional claims in published maps and institutional affiliations.

## Supplementary Material

Supplementary Information

## Figures and Tables

**Figure 1:**
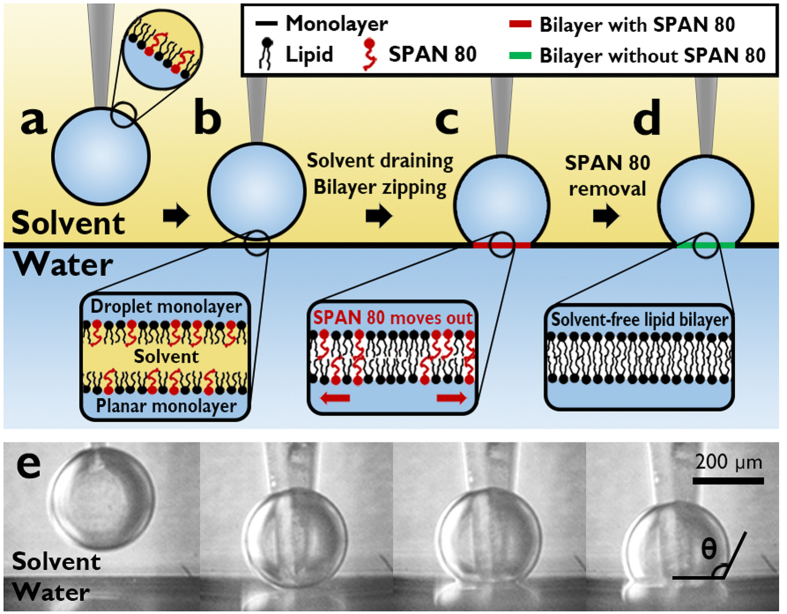
Schematics illustrating the formation of freestanding bilayer with enhanced stability and the side-view microscope images. (**a**) A lipid coated water droplet in squalene (i.e. droplet monolayer) approaches to a lipid adsorbed solvent/water interface (i.e. planar monolayer). (**b**) Solvent drains out and then two monolayers start zipping to form bilayer. (**c**) During (or after) bilayer formation, SPAN 80 is moved out of the bilayer driven by the adhesion of two monolayers. (**d**) The freestanding bilayer with large area, planar geometry and solvent & SPAN 80-free is formed. (**e**) Side-view microscopy images in correspondence with each of (**a–d**). Scale bar: 200 μm.
