# Supplementary material for: Enhanced stability of freestanding lipid bilayer and its stability criteria
Source: Sci Rep. 2016 Dec 16;6:38158. doi: 10.1038/srep38158 (PMC5159868; doi:10.1038/srep38158)
Supplement: Supplementary Information [file srep38158-s1.pdf]

Enhanced stability of freestanding lipid bilayer and its stability criteria

Dae-Woong Jeong[1], Hyunwoo Jang[1], Siyoung Q. Choi[2*] and Myung Chul Choi[1*]

[1]Department of Bio and Brain engineering, KAIST, Daejeon, 305-701, Korea

[2]Department of Chemical and Biomolecular Engineering, KAIST, Daejeon, 305-701, Korea

Table of Contents

The procedure of freestanding lipid bilayer formation ……………….…………………………… S1
Monolayer interfacial tension measurement using pendant drop method ……………………… S2
The estimation of bilayer adhesion energy and entropy of mixing of SPAN 80 ……………….. S3
The procedure of water permeability measurement …………………………………………….. S4
The procedure of bilayer tension and adhesion energy measurement ………………………….. S5
References ………………………………………………………………………………………. S6

The procedure of freestanding lipid bilayer formation

Dimyristoylphosphatidylcholine (DMPC) and dioleoylphosphatidylcholine (DOPC) are purchased from Avanti Polar Lipids and SPAN 80 from Sigma-Aldrich. We use deionized water (Milli-Q) for all of our experiments. The imaging experiments were performed by using home-built side-view microscope. The sample of phospholipid (DMPC or DOPC) in chloroform is contained in glass vial and dried in vacuum. SPAN 80 dissolved in squalene is added into the dried phospholipid, and then sonicated for 30 minutes. We prepare a trough filled with water, and the phospholipid solution is placed on top of water to form a planar squalene/water interface. The glass capillary of 0.78/1.0 mm in inner/outer diameter respectively is tapered to 10 μm of diameter by a micropipette puller (P-1000, Sutter Instrument). The capillary is filled with water and then mounted to the micro-injector (Femtojet, Eppendorf). The capillary tip is placed above the squalene/water interface. By applying a pressure of ~100 hPa, the droplet of ~300 μm diameter is introduced right above the planar interface. Both planar and droplet squalene/water interfaces are incubated for over 10 minutes for the adsorption of phospholipid and SPAN 80 monolayers, which are termed as planar monolayer and droplet monolayer, respectively. The droplet is moved toward the planar interface until the droplet gently touches the planar interface. After a few minutes of waiting, two monolayers undergo "zipping" process, in result, form the lipid bilayer between two water phases. The size of freestanding lipid bilayer can be controlled by adjusting the droplet size.

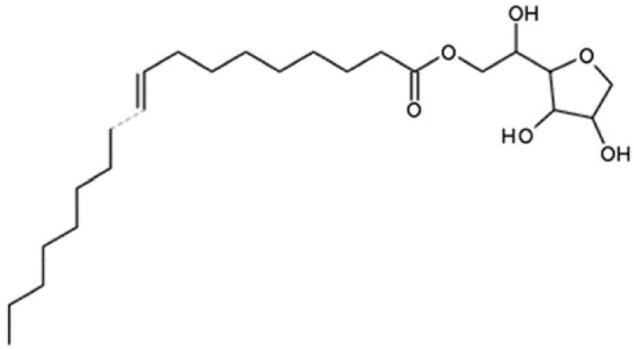

Figure S1. Chemical structure of SPAN 80[1]

Monolayer interfacial tension measurement using pendant drop method

Phospholipid solution for the monolayer interfacial tension measurements is prepared by same method as described in the procedure of freestanding lipid bilayer formation. A transparent trough is filled with the phospholipid solution and a stainless steel needle (15-gauge, OD = 1.83mm) mounted on a water filled gastight syringe is immersed. A water droplet with a volume of ~5 μL is introduced to form a solvent/water interface as shown in the Figure S2(b). Side-view images of the droplet are recorded by CCD camera (WAT-902H Ultimate, Watec) with the interval of one minute during the adsorption of phospholipids. After recording, the observed droplet contour is fitted to the Young-Laplace curve. The Bond number (β) that minimizes the mean square displacement is obtained from each image. After the fitting procedures, the interfacial tension is calculated from the formula:

$$\gamma = \frac{(\Delta\rho) g R_0^2}{\beta}$$

where $\boldsymbol{\gamma}$ is the monolayer interfacial tension, $\Delta\rho$ is the density difference between water and phospholipid solution, g is the gravitational acceleration, R_0 is the curvature radius at the apex of the droplet, and $\boldsymbol{\beta}$ is the Bond number.[2]

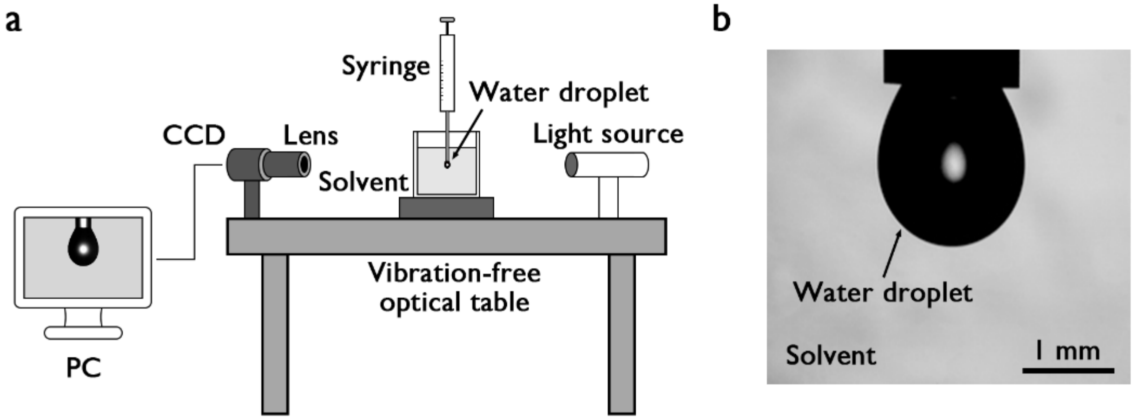

Figure S2. (a) A schematic of the pendant drop tensiometer setup. (b) Side-view image of a water droplet hanging at the end of the syringe needle.

The estimation of bilayer adhesion energy and entropy of mixing of SPAN 80

To maximize the adhesion, the SPAN 80 in the freestanding lipid bilayer moves out of the bilayer unless the effect of adhesion ($\varepsilon = \gamma_B - 2\gamma_M$, where ε is the adhesion energy, γ_B is the interfacial tension of bilayer, and γ_M is the interfacial tension of monolayer[3]) exceeds the entropic penalty, i.e. the entropy of mixing ($\Delta S = nR(x_1 ln x_1 + x_2 ln x_2)$, where n is the total number of moles, R is the gas constant, and x_i is the mole fraction of component i). Here, the entropy of mixing is always lower than nR, since the value of $x_1 ln x_1 + x_2 ln x_2$ is less than 1. Therefore, one can calculate the upper bound of the entropy of mixing as $-T\Delta S_{Max}/(area) = -nRT\ /(area) = -k_B T/A$ where A is the area per molecule, k_B is Boltzmann constant, and T is the temperature. If the adhesion exceeds the upper bound of the entropy of mixing, we can assure that the SPAN 80 is fully removed from the bilayer. Figure S3 is the plot of the adhesion energy that guarantees the removal of SPAN 80 as a function of area per SPAN 80 molecule in the lipid bilayer. For low area per SPAN 80 molecule, more adhesion is necessary to guarantee the removal of SPAN 80. For DOPC bilayer, SPAN 80 is fully removed by adhesion if the area per SPAN 80 molecule is higher than 4.07 nm^2. For DMPC bilayer, the SPAN 80 is fully removed if the area per SPAN 80 molecule is higher than 0.56 nm^2. Since these values of area per SPAN 80 molecule are plausible[4], the estimated entropic penalty of SPAN 80 is at most in the same order of magnitude of the energetic gain by introducing more DOPC or DMPC in bilayer region.

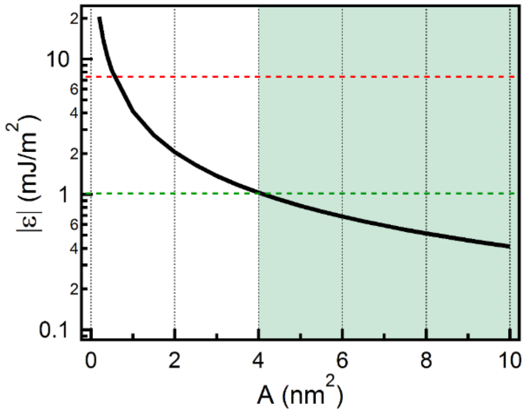

Figure S3. The adhesion energy that guarantees the removal of SPAN 80 as a function of area per SPAN 80 molecule in the lipid bilayer, where $\boldsymbol{\varepsilon}$ is the adhesion energy, $\boldsymbol{A}$ is the area per SPAN 80 molecule. The dotted red line and dotted green line indicates the adhesion energy for DMPC (-7.36 mJ/m^2) and DOPC (-1.01 mJ/m^2) bilayer respectively. The green area shows a regime of area per SPAN 80 molecule that guarantees the removal of SPAN 80 in both DMPC and DOPC bilayer.

The procedure of water permeability measurement

During fabrication of freestanding lipid bilayer for permeability measurement, aqueous solution with 100 mM NaCl is used as the subphase of planar interface instead of deionized water. Since the droplet used for the bilayer formation contains no salt, osmotic gradient is generated across the freestanding lipid bilayer. This osmotic gradient induces water transport through the freestanding lipid bilayer which results in size reduction of the droplet. The permeability of freestanding lipid bilayer is determined by tracing this size change of the droplet ($\frac{d\boldsymbol{V}(\boldsymbol{t})}{d\boldsymbol{t}} = -\boldsymbol{PS}(\boldsymbol{t})\boldsymbol{v_m}\Delta\boldsymbol{C}$, where $\boldsymbol{P}$ is the water permeability of bilayer, $\boldsymbol{V}$ is the volume of droplet, $\boldsymbol{S}$ is the area of bilayer, $\boldsymbol{v_m} = \mathbf{18\ mL/mol}$ is the water molar volume, and $\Delta\boldsymbol{C} = \mathbf{100\ mM}$ is the concentration difference).[3]

Here, the volume of droplet and the area of bilayer is measured from side-view microscope images as shown in the Figure S4. We firstly put five reference points manually at the surface of droplet and put other two points at the planar interface. Using these points, the surface of droplet and the planar interface are fitted to ellipse and straight line respectively. We assume that the volume of droplet is same with the volume of spheroid obtained by rotating the fitted ellipse above the planar interface. The half-length of planar interface inside the fitted ellipse is considered as the radius of bilayer and the area of bilayer is calculated as the area of circle with the measured radius.

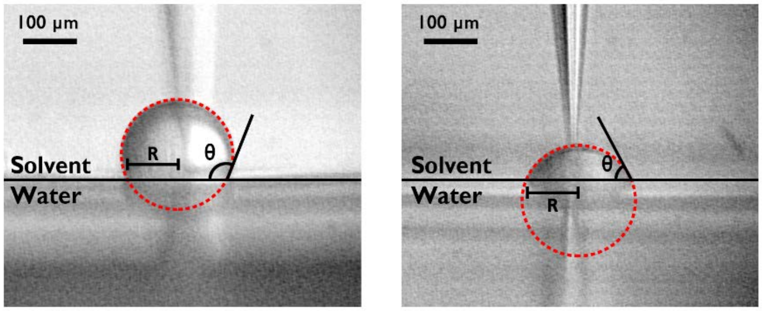

Figure S4. Side-view microscopy images of freestanding lipid bilayer for DOPC (left) and DMPC (right) with fitted lines (dotted red ellipse for droplet, solid dark line for planar interface).

The procedure of bilayer tension and adhesion energy measurement

The bilayer interfacial tension is determined to balance with the interfacial tension of the droplet and planar monolayer. The values of droplet and planar monolayer interfacial tension at certain times are estimated by interpolation of time-dependent monolayer interfacial tension data measured by the pendant drop method. The contact angle of the droplet is measured as the internal angle at the crossing point of the surface of droplet and planar interface (see Figure S4). The bilayer tension is calculated from Young's equation ($\gamma_B = \gamma_{M\cdot planar} - \gamma_{M\cdot droplet} \cdot \cos\theta$, where γ_B is the bilayer interfacial tension, $\gamma_{M\cdot planar}$ is the interfacial tension of planar monolayer, $\gamma_{M\cdot droplet}$ is the interfacial tension of droplet monolayer, and θ is the contact angle.).[3] The adhesion energy ($\varepsilon = \gamma_B - \gamma_{M\cdot planar} - \gamma_{M\cdot planar}$, where ε is the adhesion energy) is calculated using the result of bilayer interfacial tension measurement.

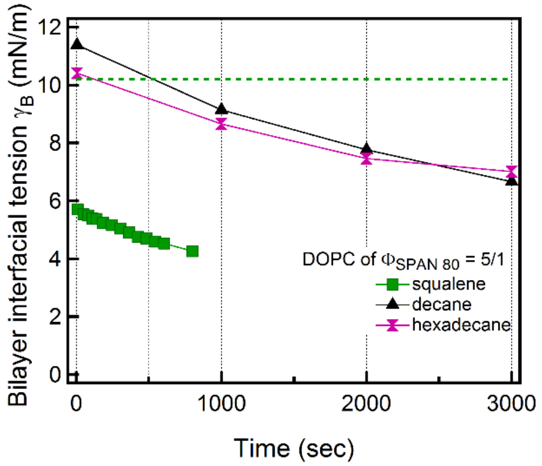

Figure S5. The bilayer interfacial tension vs. time of freestanding lipid bilayer for DOPC and DMPC at $\Phi_{SPAN\ 80}$ = 5/1 for different solvents. The dotted lines indicates the rupture tension of DOPC bilayer from Hunter et al., 1998.

References

(1) Ciriminna, R., Pantaleo, G., Mattina, R. L. & Pagliaro M. Thermogravimetric investigation of sol–gel microspheres doped with aqueous glycerol. Sustain. Chem. Proc. 2, 26 (2014)

(2) Alvarez, N. J., Walker, L. M. & Anna, S. L. A non-gradient based algorithm for the determination of surface tension from a pendant drop: Application to low Bond number drop shapes. *J. Colloid Interface Sci.* **333**, 557-562 (2009).

(3) Thiam, A., Bremond, N., & Bibette, J. From stability to permeability of adhesive emulsion bilayers. *Langmuir* **28**, 6291-6298 (2012).

(4) Apenten, R. K. O. & Zhu, Q.-H. Interfacial parameters for selected Spans and Tweens at the hydrocarbon—water interface. *Food Hydrocolloids* **10**, 27-30 (1996).
